# American Academy of Dental Science—Resolutions of Respect to Dr. W. W. Allport and Dr. E. N. Harris

**Published:** 1893-06

**Authors:** 


### Obituary.

### AMERICAN ACADEMY OF DENTAL SCIENCE—RESOLUTIONS OF RESPECT TO DR. W. W. ALLPORT AND DR. E. N. HARRIS.

*Resolved*, That by the decease of Dr. W. W. Allport, of Chicago, the American Academy of Dental Science loses from its list of honorary members one who with unfailing integrity has represented during his long professional life the highest qualities as a sound and progressive practitioner during a most remarkable period in our profession.

*Resolved*, That we deem his removal a loss not only to ourselves, but to the whole profession in this country and all the civilized world.

*Resolved*, That this expression be recorded and a copy be sent to his family.

JACOB L. WILLIAMS,
THOS. FILLEBROWN,
R. R. ANDREWS,
*Committee.*

*Resolved*, That the American Academy of Dental Science learns with sorrow of the decease of Dr. E. N. Harris, an original member who has given his constant service to the Academy since its formation, during twenty-five years.

*Resolved*, That his faithful, conscientious, and unremitting labors are deeply appreciated and will be long remembered by his associates in the Society.

*Resolved*, That the above be placed on record and a copy be sent to his family.

JACOB L. WILLIAMS,
R. R. ANDREWS,
THOS. FILLEBROWN,
*Committee.*

